# Isolation and biosynthesis of daturamycins from *Streptomyces* sp. KIB-H1544

**DOI:** 10.3762/bjoc.18.101

**Published:** 2022-08-09

**Authors:** Yin Chen, Jinqiu Ren, Ruimin Yang, Jie Li, Sheng-Xiong Huang, Yijun Yan

**Affiliations:** 1 State Key Laboratory of Phytochemistry and Plant Resources in West China, and CAS Center for Excellence in Molecular Plant Sciences, Kunming Institute of Botany, Chinese Academy of Sciences, Kunming 650201, China; 2 University of Chinese Academy of Sciences, Beijing 100049, China

**Keywords:** biosynthesis, diarylcyclopentenone, polyporic acid synthetase, *p*-terphenyl, *Streptomyces*

## Abstract

Two novel diarylcyclopentenones daturamycin A and B (**1** and **2**), and one new *p*-terphenyl daturamycin C (**3**), along with three known congeners (**4**–**6**), were isolated from a rhizosphere soil-derived *Streptomyces* sp. KIB-H1544. The structures of new compounds were elucidated via a joint use of spectroscopic analyses and single-crystal X-ray diffractions. Compounds **1** and **2** belong to a rare class of tricyclic 6/5/6 diarylcyclopentenones, and compounds **3**–**6** possess a C-18 tricyclic aromatic skeleton. The biosynthetic gene cluster of daturamycins was identified through gene knockout and biochemical characterization experiments and the biosynthetic pathway of daturamycins was proposed.

## Introduction

Natural products containing a terphenyl skeleton exhibit a large number of structural diversity due to the differences of the center ring and the connection among rings. Structurally, most natural terphenyls are *p*-terphenyl derivatives consisting of a C-18 tricyclic or polycyclic C-18 aromatic skeleton. Diarylcyclopentenones, which possess a rare class of a tricyclic 6/5/6 system, could also belong to *p*-terphenyl derivatives from a biosynthetic perspective. To date, more than 230 natural products containing *p*-terphenyl have been unearthed in nature, among them many *p*-terphenyls were isolated from fungi, only a few were discovered from *Streptomyces* species [1–4]. Meanwhile, these types of compounds exhibit a broad spectrum of bioactivities, including antitumor [5–6], antibacterial [7–8], antioxidant [9–12], immunosuppressive [13], and antithrombotic activities [14].

**Figure 1:**
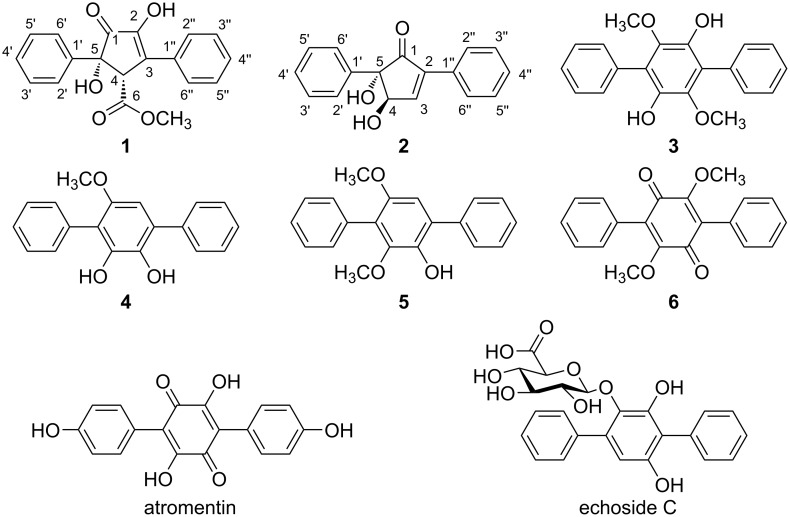
Structures of compounds **1**–**6**, atromentin, and echoside C.

The biosynthesis of the *p*-terphenyls has been studied since the 1960s [15–16]. Stable-isotope labeling experiments confirmed that ʟ-tyrosine or ʟ-phenylalanine are involved in the biosynthesis of *p*-terphenyl as metabolic origin. The precursors undergoing deamination are converted to the corresponding α-keto acid, then a quinone intermediate arises by condensation between two molecules of α-keto acid. Structurally diverse *p*-terphenyls are formed from these key intermediates by several tailoring reactions such as cyclization, tautomerization, methylation, and glycosylation. A previous study has shown that the formation of 2,5-diarylcyclopentenone proceeds via the terphenylquinone atromentin (Figure 1), followed by oxidative ring contraction [17]. However, the details of cyclopentenone formation involving ring contraction have remained unclear.

In this study, two novel diarylcyclopentenones daturamycins A amd B (**1** and **2**), one new *p*-terphenyl daturamycin C (**3**), and three known *p*-terphenyl derivatives (**4**–**6**) (Figure 1) were isolated from the fermentation extract of *Streptomyces* sp. KIB-H1544. Structurally, daturamycins A and B are uncommon diarylcyclopentenone compounds that consist of a tricyclic 6/5/6 system instead of a C-18 tricyclic skeleton in other compounds. To explore the biosynthesis of daturamycins in *S*. sp. KIB-H1544, especially the formation mechanism of the tricyclic 6/5/6 scaffold, the biosynthetic gene cluster of daturamycins was found and confirmed by gene knockout experiments. We also characterized the deduced peptide synthetase DatA, which catalyzes the Claisen–Dieckmann condensation of phenylpyruvic acid (**7**) to generate the key intermediate polyporic acid (**8**). Finally, we proposed a biosynthetic pathway for daturamycins.

## Results and Discussion

**Figure 2:**
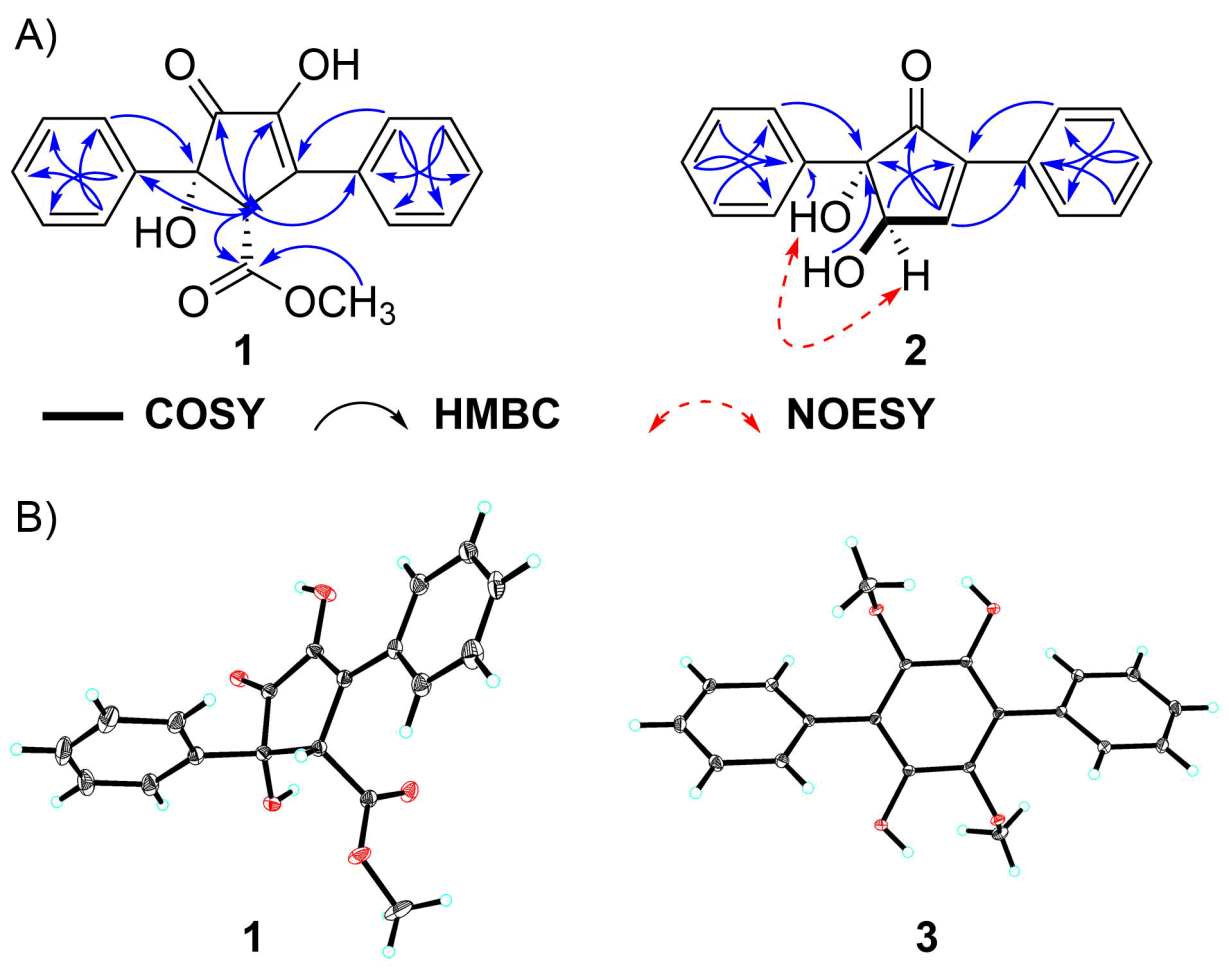
(A) Key 2D NMR correlations of compounds **1** and **2**. (B) X-ray crystal structure of compounds **1** and **3**.

Daturamycin A (**1**), a yellow powder, possessed a molecular formula of C_19_H_16_O_5_ with 12 degrees of unsaturation based on the HRMS–ESI data (*m/z* 347.0893 [M + Na]^+^, calcd for C_19_H_16_O_5_Na^+^, 347.0890) (Figure S1, Supporting Information File 1). Comprehensive analysis of the ^1^H and ^13^C NMR data (Table 1, Figures S3 and S4, Supporting Information File 1) and HSQC data (Figure S5, Supporting Information File 1) demonstrated the presence of one methyl, eleven methines (including ten aromatic ones), and seven quaternary carbons (including two carbonyl carbons). The structural information of **1** was further defined according to HMBC data (Figure S6, Supporting Information File 1). The key HMBC signals (H-2’ to C-1’, C-4’, C-6’; H-3’ to C-1’, C-5’; H-4’ to C-2’, C-6’ ; H-5’ to C-1’, C-3’; H-2’’ to C-1’’, C-4’’, C-6’’; H-3’’ to C-1’’, C-5’’; H-4’’ to C-2’’, C-6’’, and H-5’’ to C-1’’, C-3’’) of compound **1** indicated the presence of two mono-substituted aromatic rings. The cross-peaks from H-4 to C-1, C-2, C-3, C-5, and C-6, from 6-OCH_3_ to C-6 established a central five-membered carbon ring. Meanwhile, the correlations from H-4 to C-1’, C-1’’, from H-2’ and H-6’ to C-5, and from H-2’’ and H-6’’ to C-3 showed that two benzene rings were conjugated to the central ring at the C-3 and C-5 positions (Figure 2). The NMR data indicated that compound **1** shares a similar skeleton to the known compound (±)-tylopilusin B [18]. The additional signals suggested that three methines (δ_C_/δ_H_ 59.2/4.49, CH-4; 129.0/7.27, CH-4’; 130.5/7.38, CH-4’’) in compound **1** were hydroxylated in (±)-tylopilusin B. Furthermore, the absolute configuration of C-4 and C-5 in compound **1** was also confirmed as 4*R* and 5*S* by X-ray crystallography (Figure 2).

**Table 1:** ^1^H (600 MHz) and ^13^C (150 MHz) NMR data of compounds **1** and **2**.

	**1** (in CD_3_OD)		**2** (in DMSO-*d*_6_)	
	
position	δ_C_	δ_H,_ mult. (*J* in Hz)	δ_C_	δ_H,_ mult. (*J* in Hz)

1	201.2, C		204.6, C	
2	151.4, C		140.8, C	
3	135.1, C		157.7, CH	7.98, d (1.8)
4	59.2, CH	4.49, s	77.5, CH	4.84, d (4.3)
5	79.4, C		85.5, C	
6	172.8, C			
1’	143.5, C		139.6, C	
2’/6’	126.2, CH	7.44, d (7.7)	126.9, CH	7.28, m
3’/5’	129.5, CH	7.33, t (7.5)	127.3, CH	7.28, m
4’	129.0, CH	7.27, t (7.3)	126.7, CH	7.21, t (6.9)
1’’	134.7, C		130.7, C	
2’’/6’’	128.7, CH	7.95, d (7.7)	127.1, CH	7.82, d (7.3)
3’’/5’’	129.8, CH	7.44, t (7.5)	128.6, CH	7.45, t (7.3)
4’’	130.5, CH	7.38, t (7.3)	129.0, CH	7.41, t (7.3)
6-OCH_3_	52.9, CH_3_	3.71, s		
4-OH				5.60, d (6.3)
5-OH				6.29, s

(±)-Daturamycin B (**2**) was isolated as a white powder, and its molecular formula was determined as C_17_H_14_O_3_ by HRMS–ESI data (*m/z* 289.0833 [M + Na]^+^, calcd for C_19_H_16_O_5_Na^+^, 289.0835) (Figure S7, Supporting Information File 1), and the unsaturation was 11 degree. It could be deduced that compound **2** also possesses a similar core to compound **1**, based on the molecular formula and NMR data (Table 1, Figures S8–S13, Supporting Information File 1). This could be confirmed by the key COSY correlation of H-3/H-4 and the HMBC correlations (H-3 to C-1, C-4, C-1’’; H-4 to C-1, C-2, C-1’; 4-OH to C-3, C-5; 5-OH to C-1, C-4, C-1’; H-2’ to C-5; H-3’ to C-1’; H-4’ to C-2’, C-6’; H-2’’ to C-2, C-3, C-4’’; H-3’’ to C-1’’; H-4’’ to C-2’’). The NOESY correlation (Figure 2) between H-4 and 5-OH suggested that the relative configurations of C-4 and C-5 were *trans*. Therefore, the structure of compound **2** has been determined, as shown in Figure 1. However, the absolute configuration of compound **2** remained unsolved.

Daturamycin C (**3**) was obtained as a brown powder, and the ^1^H and ^13^C NMR spectra (Figures S14 and S15, Supporting Information File 1) indicated that compound **3** showed strong similarity with 1,4-diphenyl-2,3,5,6-tetramethoxybenzene [19], with two methyl signals were absent in the NMR data of **3**. Furthermore, the structure of **3** could be confirmed, as shown in Figure 1, based on X-ray crystallography (Figure 2).

The known congeners were determined as terferol (**4**) [20], BTH-II0204-207: D (**5**) [21], and betulinan A (**6**) [22] (Figure 1) by comparing their ^1^H and ^13^C NMR spectroscopic data (Figures S16–S21, Supporting Information File 1) and specific rotation values with those in the literatures.

**Figure 3:**
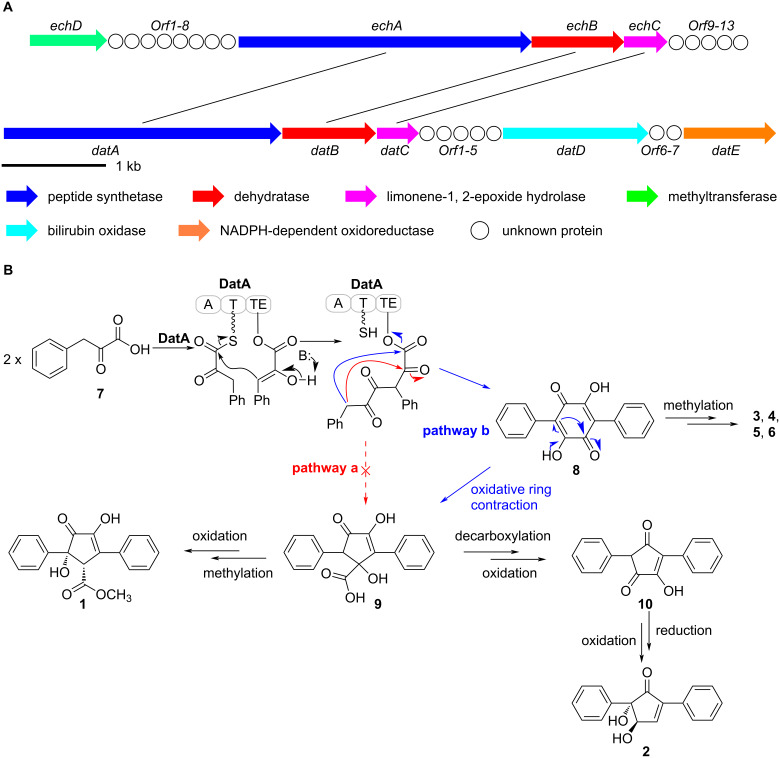
(A) The biosynthetic gene cluster of daturamycins. (B) Proposed biosynthetic pathway of daturamycins.

Diarylcyclopentenones, characteristic constituents of mushrooms [23], were rarely discovered in *Streptomyces* species. These components exhibit redox activity and are involved in reducing ferric (Fe^3+^) in the Fenton-based biological decomposition of lignocellulose [24–25]. The biosynthetic pathway of *p*-terphenyl was previously identified in *Paxillus involutus*, and atromentin was supposed to be a metabolic precursor of diarylcyclopentenone [17]. However, the mechanism of cyclopentenone moiety formation has remained unclear. Two pathways may be involved in the biosynthesis of diarylcyclopentenone (Figure 3B): (path a) two molecules of phenylpyruvic acid (**7**) undergo direct condensation to form a five-membered ring intermediate or (path b) polyporic acid (**8**) undergoes oxidative ring contraction or conversion to generate the cyclopentenone skeleton.

**Figure 4:**
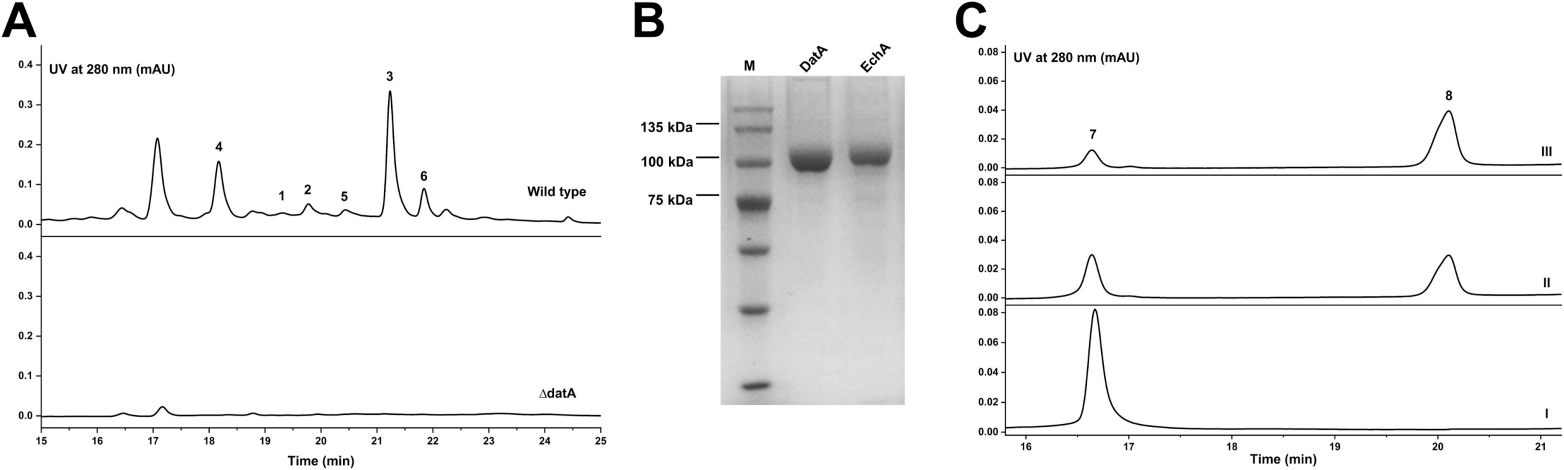
(A) HPLC analysis of the fermentation extracts of mutant *S*. sp. KIB-H1544-*∆datA*. (B) SDS-PAGE analysis of purified DatA (calculated molecular mass 101.8 kDa) and EchA (calculated molecular mass 105.4 kDa) protein. (C) HPLC analysis of *in vitro* enzyme reactions. Panel I, phenylpyruvic acid with boiled DatA; panel II, phenylpyruvic acid with EchA; panel III, phenylpyruvic acid with DatA.

To verify which pathway was responsible for the biosynthesis of daturamycins in *S.* sp. KIB-H1544, we conducted the following experiments. Firstly, the genome of *S.* sp. KIB-H1544 was sequenced, and bioinformatic analysis yields the three-genes cluster (NCBI accession number: ON973849) encoded by *datA* (peptide synthetase), *datB* (NAD-dependent dehydratase), and *datC* (limonene-1,2-epoxide hydrolase), which share high sequence similarity with the echoside (Figure 1) biosynthetic gene cluster [26] (EchA, 69.2% identity to DatA; EchB, 78.9% identity to DatB; EchC, 66.9% identity to DatC) (Figure 3A). Additionally, one bilirubin oxidase (DatD) and NADPH-dependent oxidoreductase (DatE) are found downstream of the three-genes cluster (Table S1, Supporting Information File 1). Subsequently, we set out to knockout *datA* gene by λ-RED-mediated recombination in *S.* sp. KIB-H1544. Culture and fermentation results of mutant *S*. sp. KIB-H1544-*∆datA* indicated that the primary production of compounds **1**–**6** in the mutant was eliminated (Figure 4A). This result suggested that the *datA* gene is responsible for the biosynthesis of daturamycins. Lastly, in order to verify the cyclopentenone rings of daturamycins A and B are generated via direct condensation of phenylpyruvic acid (**7**) catalyzed by DatA, the DatA protein was expressed and purified (Figure 4B). Incubating DatA with substrate **7** and ATP resulted in a new product **8**, which was confirmed by HRMS–ESI data (*m/z* 291.0663 [M − H]^−^, calcd for ([C_18_H_12_O_4_] − H)^−^, 291.0663) (Figure S22, Supporting Information File 1). The product was not present in the control reaction with boiled DatA protein (Figure 4B). Furthermore, we could not detect any other intermediates in the reaction mixture through MS analysis (Figure S23, Supporting Information File 1). These results suggested that DatA could not catalyze the formation of the cyclopentenone ring. It has the same function as EchA, which only catalyzes the Claisen–Dieckmann condensation of phenylpyruvic acid (**7**) to generate polyporic acid (**8**) [26].

Based on the gene knockout and biochemical characterization experiments, we proposed a possible biosynthetic pathway for daturamycins in *S.* sp. KIB-H1544 (Figure 3B). First, two molecules of phenylpyruvic acid (**7**), which is deaminated from ʟ-phenylalanine, are condensed into the six-membered polyporic acid (**8**) catalyzed by DatA. Then, **8** converts to compounds **3**, **4**, **5**, and **6** through methylation and oxidation. On the other hand, polyporic acid (**8**) probably undergoes rearrangement, decarboxylation, methylation reaction, and further modification to generate compounds **1** and **2**.

## Conclusion

*S.* sp. KIB-H1544 was isolated from the rhizosphere soil of *Datura stramonium* L. Two novel diarylcyclopentenones daturamycins A and B and one new *p*-terphenyl daturamycin C, were isolated and identified. The new diarylcyclopentenones possess a rare class of a tricyclic 6/5/6 system. Based on the bioinformatics analysis, the daturamycins biosynthetic gene cluster has been identified and confirmed by the inactivation of the structural gene *datA*. The following biochemical characterization indicated that the DatA is a polyporic acid synthetase, which helped to propose a possible biosynthetic pathway for daturamycins in S. sp. KIB-H1544 (Figure 3B). Finally, the discovery of daturamycins, identification, and characterization of *dat* biosynthetic gene cluster allowed us to explore their biosynthesis, especially the mechanism of cyclopentenone formation in the diarylcyclopentenones.

## Experimental

### General experimental procedures

IR spectra were measured on a Bruker Tensor 27 FTIR spectrometer using KBr disks. NMR spectra were performed with a Bruker AV600 MHz spectrometer with TMS as an internal standard. HRMS–ESI data were obtained through an Agilent 1290 UPLC/6540 Q-TOF mass instrument. Column chromatography (CC) was performed using silica gel (30–400 mesh, Qingdao Marine Chemical Inc., China), Sephadex LH-20 (25–100 µm, Pharmacia Biotech Ltd., Sweden), and MCI gel (75–150 µm, Mitsubishi Chemical Corporation, Tokyo, Japan). Semipreparative HPLC was conducted on a HITACHI Chromaster system equipped with a DAD detector, a YMC-Triart C18 column (250 × 10 mm, i.d. 5 μm), and a flow rate of 3.0 mL/min. LC–MS was performed using an Agilent 1200 series HPLC system coupled to an Agilent q TOF 6540 mass spectrometer with a YMC-Triart C18 column (250 × 4.6 mm, i.d. 5 μm) and a ﬂow rate of 1.0 mL/min.

### Extraction and isolation

*S.* sp. KIB-H1544, isolated from the rhizosphere soil of *Datura stramonium* L., was 99.9% similar to the 16S rRNA of *Streptomyces roseofulvus* NBRC15816. The strain was cultured in 20 L M15 medium: 30.0 g/L glucose, 1.0 g/L peptone, 5.0 g/L beef paste, 2.5 g/L CaCO_3_, 5.0 g/L NaCl, 1.0 mL/L trace elements solution (1.0 g/L FeSO_4_·7H_2_O, 0.45 g/L CuSO_4_·5H_2_O, 1.0 g/L ZnSO_4_·7H_2_O, 0.1 g/L MnSO_4_·4H_2_O, 0.1 g/L K_2_MoO_4_). After extraction and concentration, the extract was purified by silica gel chromatography and semipreparative HPLC to yield daturamycin A (**1**) (4.5 mg), daturamycin B (**2**) (1.8 mg), daturamycin C (**3**) (8.7 mg), terferol (**4**) (2.4 mg), BTH-II0204-207: D (**5**) (2.1 mg), betulinan A (**6**) (6.2 mg).

### Identification of new compounds

**Daturamycin A** (**1**): pale yellow solid; IR (KBr) ν_max_: 3468, 3287, 1731, 1702, 1631, 1398 and 1202 cm^−1^; UV (MeOH) λ_max_ (log ε): 314 (0.48), 217 (0.28), 202 (0.48); ^1^H and ^13^C NMR, see Table 1; HRMS–ESI (*m*/*z*): [M + Na]^+^ calcd for C_19_H_16_O_5_Na^+^, 347.0890; found, 347.0893.

**Daturamycin B** (**2**): white powder; ^1^H and ^13^C NMR, see Table 1; HRMS–ESI (*m*/*z*): [M + Na]^+^ calcd for C_19_H_16_O_5_Na^+^, 289.0835; found, 289.0833.

**Daturamycin C** (**3**): brown powder; ^1^H NMR (600 MHz, CDCl_3_) δ 7.57 (d, *J* = 7.2 Hz, 4H), 7.48 (t, *J* = 7.6 Hz, 4H), 7.39 (t, *J* = 7.4 Hz, 2H), 5.52 (s, 2H), 3.38 (s, 6H); ^13^C NMR (150 MHz, CDCl_3_) δ 140.9, 139.6, 132.8, 130.6, 130.3, 128.3, 128.0, 127.7, 120.7.

**X-ray crystal data of compound 1:** C_19_H_16_O_5_, *M* = 324.32, *a* = 9.8765(14) Å, *b* = 8.9786(13) Å, *c* = 18.315(3) Å, α = 90°, β = 92.119(3)°, γ = 90°, *V* = 1623.0(4) Å^3^, *T* = 100(2) K, space group *P*21/*n*, *Z* = 4, μ(Mo Kα) = 0.096 mm^−1^, 16091 reflections measured, 4037 independent reflections (Rint = 0.0728). The final R1 values were 0.0526 (I > 2σ(I)). The final wR(F2) values were 0.1003 (I > 2σ(I)). The final R1 values were 0.1061 (all data). The final wR(F2) values were 0.1183 (all data). The goodness of fit on F2 was 1.003.

**X-ray crystal data of compound 3:** C_20_H_18_O_4_, *M* = 322.34, *a* = 5.9209(8) Å, *b* = 20.882(3) Å, *c* = 7.0856(10) Å, α = 90°, β = 113.939(2)°, γ = 90°, *V* = 800.70(19) Å^3^, *T* = 100(2) K, space group *P*21/*c*, *Z* = 2, μ(Mo Kα) = 0.093 mm^−1^, 8836 reflections measured, 2398 independent reflections (Rint = 0.0250). The final R1 values were 0.0423 (I > 2σ(I)). The final wR(F2) values were 0.1059 (I > 2σ(I)). The final R1 values were 0.0495 (all data). The final wR(F2) values were 0.1101 (all data). The goodness of fit on F2 was 1.051.

### Genomic library construction and screening

The genome of *S*. sp. KIB-H1544 was sequenced through the Illumina Genome Analyzer (Illumina, San Diego, CA) by BGI (BGI-Shenzhen, China). According to standard procedures, the genomic DNA was digested with *Mbo*I, and the 30–42 kb DNA fragments were isolated and ligated to cosmid pSuperCos I. MaxPlax Lambda packaging extracts were used for packaging. About 2,000 *E. coli* clones were picked and stored in 20 96-well microplates at −80 °C. The positive clone, named pSC-21A4, was verified by PCR. And primers used for screening were listed in Table S3 (Supporting Information File 1).

### Gene inactivation

Primers designed for inactivation of *datA* gene are listed in Table S3 (Supporting Information File 1). The deletion mutant was constructed by λ-RED-mediated PCR targeting mutagenesis method [27]. The positive cosmid pSC-21A4 was transformed into *E. coli* BW25113/pIJ790 for gene inactivation. Then, the PCR fragment was introduced via electro-transformation into *E. coli* BW25113/pIJ790-pSC-21A4, in which the target gene *datA* would be replaced with the cassette. This recombinant cosmid was transformed into *E. coli* ET12567/pUZ8002, and suffered from intergeneric conjugation with *S*. sp. KIB-H1544 wild strain. *E. coli*-*Streptomyces* conjugation was performed on MS solid medium freshly supplemented with 10 mM MgCl_2_. Double crossover mutants were selected based on the Kan-Apr and then confirmed by PCR using primers. Finally, the mutant strain *S*. sp. KIB-H1544-*∆datA* was generated (Table S2, Supporting Information File 1).

### Expression and purification of recombinant DatA and EchA protein

The DNA fragment containing *datA* was amplified by the primer pair Duet-DatA-F/Duet-DatA-R (Table S3, Supporting Information File 1), then cleaved by *BamH* I and *Sac* I, and inserted into the corresponding site of pETDuet-*sfp* to produce plasmid pETDuet-*sfp*-*datA.* As a positive control, the DNA fragment containing *echA* gene (GenBank: KJ156360.1) was synthesized by GENEWIZ company*,* plasmid pETDuet-*sfp*-*echA* was constructed using the same method as DatA expression vector construction. The recombinant plasmids were then transformed into *E. coli* BL21 (DE3), respectively. pETDuet-*sfp*-*datA* and pETDuet-*sfp*-*echA* were grown in Luria Bertani (LB) supplemented with 100 μg/mL ampicillin at 37 °C until OD_600_ reached 0.6. IPTG (0.2 mM) was added to the final concentration of 0.4 mM and incubated at 16 °C overnight. The cells were centrifuged at 4000 rpm for 25 min and resuscitated with lysis buffer (buffer A: 50 mM Tris-HCl, 300 mM NaCl, 20 mM imidazole, pH 8.0). After ultrasonic cell crushing, the cells were centrifuged at 24,000 rpm for 60 min to remove cell fragments. The supernatant was filtered through 0.22 μm of the filter membrane and then loaded into the nickel column of the rebalanced lysate (HisTraqTM FF, GE Healthcare). The eluent was removed with buffer B (50 mM Tris-HCl, 300 mM NaCl, 250 mM imidazole, pH 8.0) at a flow rate of 2 mL/min, starting from the fourth tube of the collection tube and ending at the sixth tube. The outgoing protein was collected and poured into a precooled filter column (Ultracel series 10 KDa; GE Healthcare). Then, the collected DatA (or EchA) protein was concentrated and buffer-exchanged into storage buffer (50 mM NaH_2_PO_4_, 100 mM NaCl, pH 8.0, containing 10% glycerin). The proteins were tested by SDS-PAGE, frozen using liquid nitrogen, and kept at −80 °C.

### In vitro reactions

DatA enzymatic activity was tested in a 100 μL reaction mixture containing 100 mM Tris-HCl (pH 7.5), 10 mM MgCl_2_, 300 mM NaCl, 5 mM ATP, 0.5 mM phenylpyruvic acid and 5 μM DatA (or EchA), at 30 °C for 2 h. The reaction mixture was quenched by adding 200 μL methanal and then centrifuged at 12,000 rpm for 10 min. The supernatant was analyzed by HPLC.

## Supporting Information

File 1 Spectroscopic data for compounds **1**–**3**, HRMS–ESI data for compound **8**, annotation of genes in the *dat* biosynthetic gene cluster, list of biological material, vectors, and primers used in this study.
