# Supplementary material for: Isolation and biosynthesis of daturamycins from Streptomyces sp. KIB-H1544
Source: Beilstein J Org Chem. 2022 Aug 9;18:1009–16. doi: 10.3762/bjoc.18.101 (PMC9379647; doi:10.3762/bjoc.18.101)
Supplement: File 1 — Spectroscopic data for compounds 1–3, HRMS–ESI data for compound 8, annotation of genes in the dat biosynthetic gene cluster, list of biological material, vectors, and primers used in this study. [file Beilstein_J_Org_Chem-18-1009-s001.pdf]

BEILSTEIN JOURNAL OF ORGANIC CHEMISTRY

Supporting Information

for

Isolation and biosynthesis of daturamycins from *Streptomyces* sp. KIB-H1544

Yin Chen, Jinqiu Ren, Ruimin Yang, Jie Li, Sheng-Xiong Huang and Yijun Yan

Beilstein J. Org. Chem. **2022,** *18,* 1009–1016. doi:10.3762/bjoc.18.101

Spectroscopic data for compounds 1–3, HRMS–ESI data for compound 8, annotation of genes in the *dat* biosynthetic gene cluster, list of biological material, vectors, and primers used in this study

License and Terms: This is a supporting information file under the terms of the Creative Commons Attribution License (https://creativecommons.org/licenses/by/4.0). Please note that the reuse, redistribution and reproduction in particular requires that the author(s) and source are credited and that individual graphics may be subject to special legal provisions.
The license is subject to the *Beilstein Journal of Organic Chemistry* terms and conditions: (https://www.beilstein-journals.org/bjoc/terms)

Table of contents

Figure S1: HRMS–ESI spectrum of compound **1**. S2

Figure S2: IR spectrum of compound **1** in MeOH. S2

Figure S3: ^{1}H NMR spectrum of compound **1** (600 MHz, CD_3OD). S3

Figure S4: ^{13}C NMR spectrum of compound **1** (150 MHz, CD_3OD). S3

Figure S5: HSQC spectrum of compound **1** (600 MHz, CD_3OD). S4

Figure S6: HMBC spectrum of compound **1** (600 MHz, CD_3OD). S4

Figure S7: HRMS–ESI spectrum of compound **2**. S5

Figure S8: ^{1}H NMR spectrum of compound **2** (600 MHz, DMSO-d_6). S5

Figure S9: ^{13}C NMR spectrum of compound **2** (150 MHz, DMSO-d_6). S6

Figure S10: HSQC spectrum of compound **2** (600 MHz, DMSO-d_6). S6

Figure S11: ^{1}H-^{1}H COSY spectrum of compound **2** (600 MHz, DMSO-d_6) S7

Figure S12: HMBC spectrum of compound **2** (600 MHz, DMSO-d_6). S7

Figure S13: NOESY spectrum of compound **2** (600 MHz, DMSO-d_6). S8

Figure S14: ^{1}H NMR spectrum of compound **3** (600 MHz, $CDCl_3$). S9

Figure S15: ^{13}C NMR spectrum of compound **3** (150 MHz, $CDCl_3$) S9

Figure S16: ^{1}H NMR spectrum of compound **4** (600 MHz, DMSO-d_6). S10

Figure S17: ^{13}C NMR spectrum of compound **4** (150 MHz, DMSO-d_6). S10

Figure S18: ^{1}H NMR spectrum of compound **5** (600 MHz, DMSO-d_6). S11

Figure S19: ^{13}C NMR spectrum of compound **5** (150 MHz, DMSO-d_6). S11

Figure S20: ^{1}H NMR spectrum of compound **6** (600 MHz, $CDCl_3$). S12

Figure S21: ^{13}C NMR spectrum of compound **6** (150 MHz, $CDCl_3$) S12

Figure S22: HRMS–ESI spectrum of compound **8**. S13

Figure S23: MS analysis of DatA reaction mixture. S14

Table S1: Annotations of ORFs in the *dat* biosynthetic gene cluster S15

Table S2: Strains and plasmids used and generated in this study. S16

Table S3: Primers used in this study. S17

Fragmentor Voltage	Collision Energy	Ionization Mode
135	0	ESI

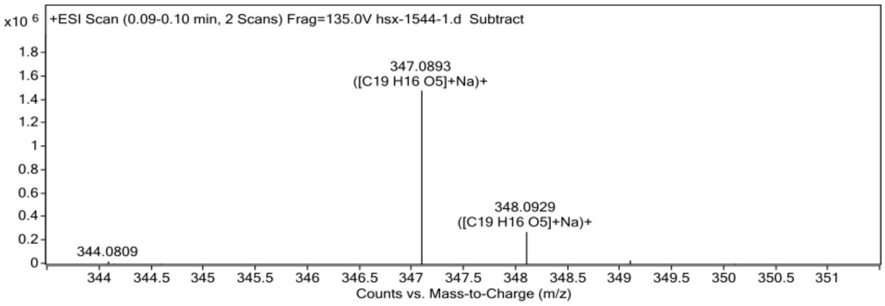

Peak List

m/z	z	Abund	Formula	Ion
247.0752	1	155253.95		
279.1016	1	152539.56		
307.0968	1	1684749.63		
308.1003	1	311080.25		
342.1341	1	351820.19		
347.0893	1	1478410.75	C19 H16 O5	(M+Na)+
348.0929	1	277042.75	C19 H16 O5	(M+Na)+
363.0633	1	213484.89		
671.1899	1	786305.25		
672.1934	1	309542.5		

Formula Calculator Element Limits

Element	Min	Max
C	3	60
H	0	120
O	0	30
N	0	10

Formula Calculator Results

Formula	CalculatedMass	CalculatedMz	Mz	Diff. (mDa)	Diff. (ppm)	DBE
C19 H16 O5	324.0998	347.0890	347.0893	-0.30	-0.86	12.0000

Figure S1: HRMS–ESI spectrum of compound **1**.

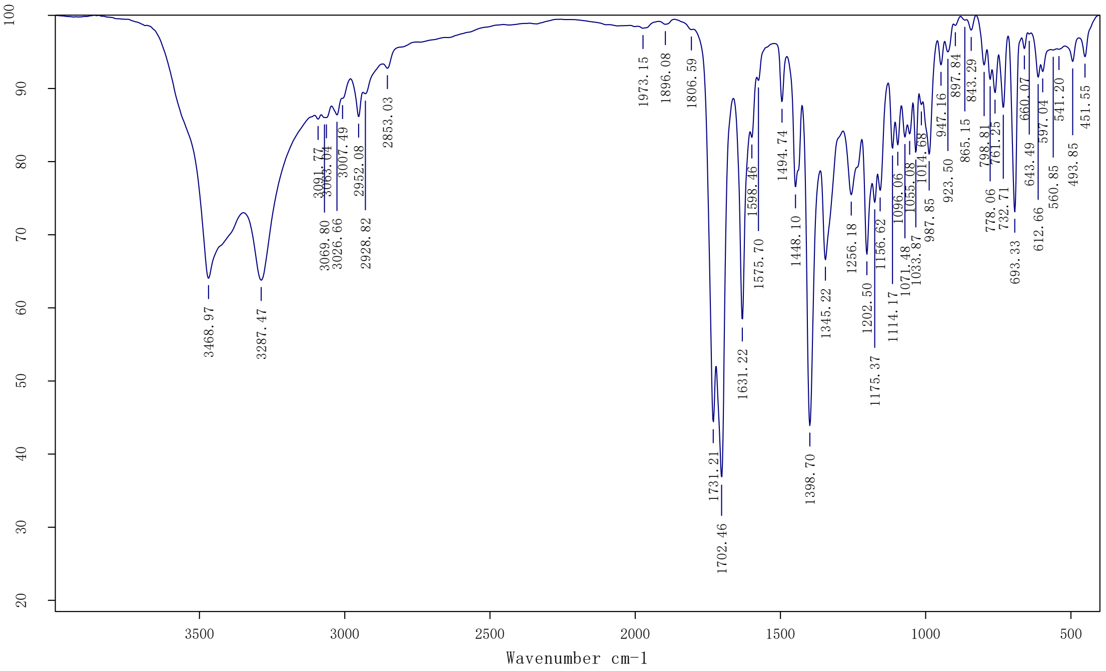

Figure S2: IR spectrum of compound **1** in MeOH.

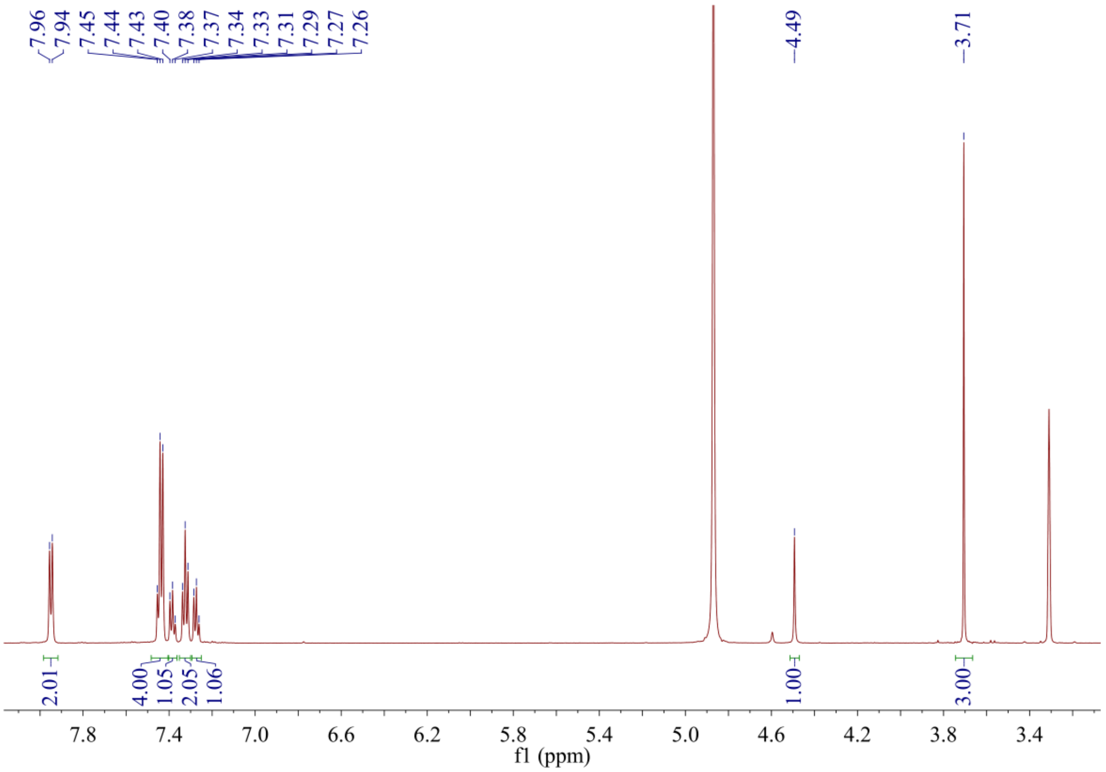

Figure S3: ^{1}H NMR spectrum of compound **1** (600 MHz, CD_3OD).

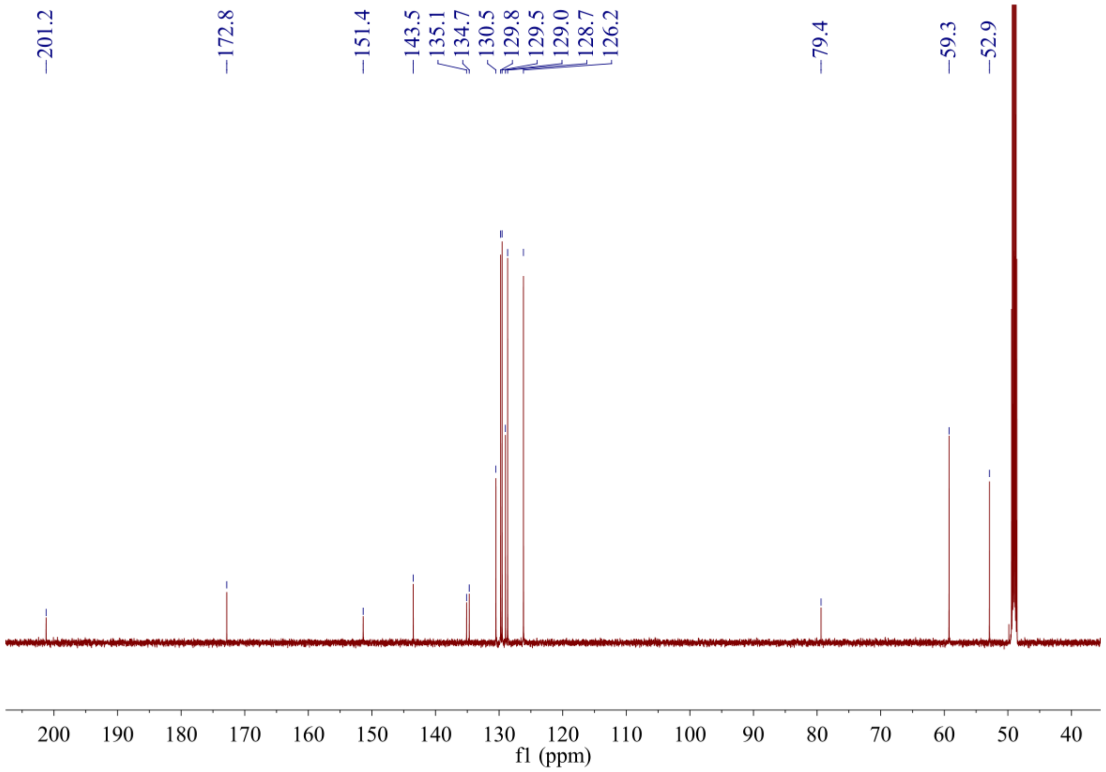

Figure S4: ^{13}C NMR spectrum of compound **1** (150 MHz, CD_3OD).

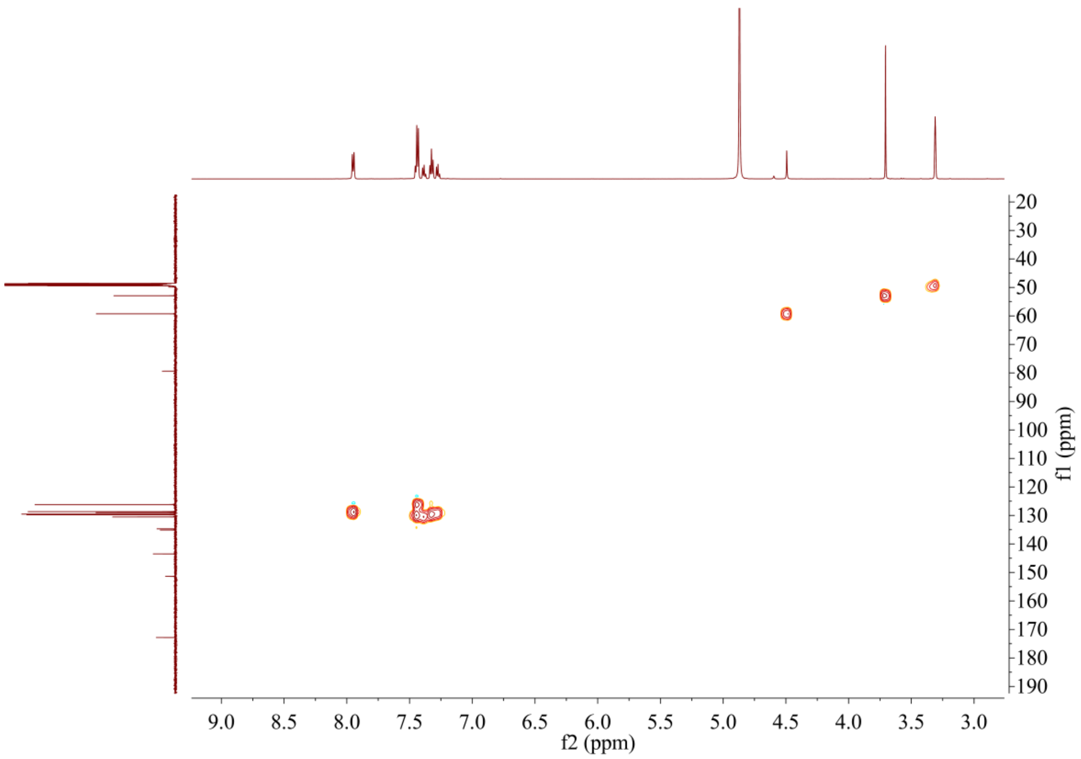

Figure S5: HSQC spectrum of compound **1** (600 MHz, CD_3OD).

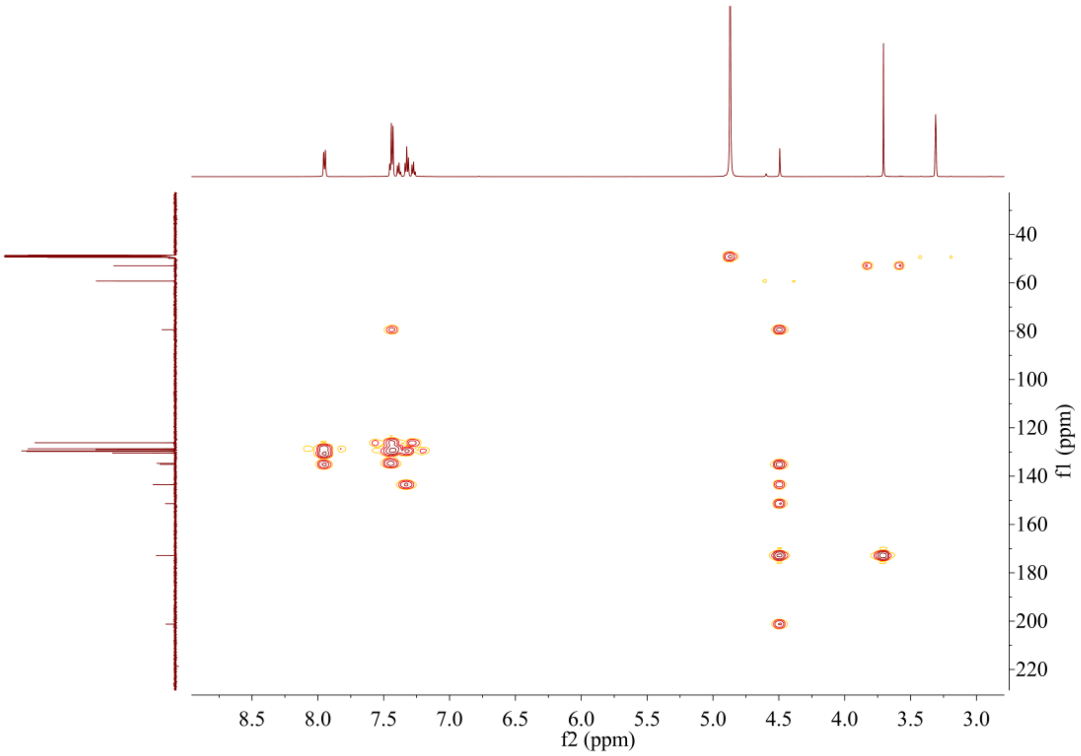

Figure S6: HMBC spectrum of compound **1** (600 MHz, CD_3OD).

Fragmentor Voltage	Collision Energy	Ionization Mode
135	0	ESI

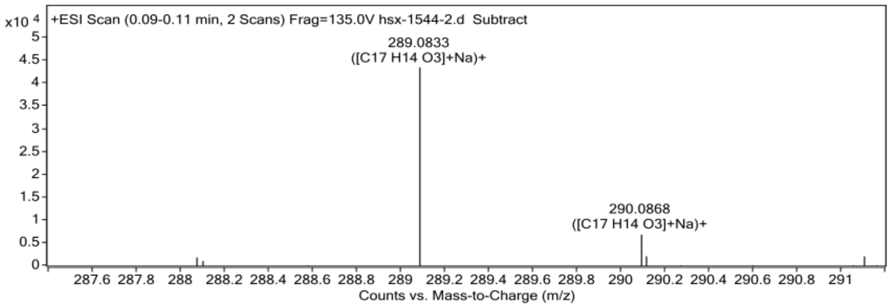

Peak List

m/z	z	Abund	Formula	Ion
105.0341	1	30080.09		
221.0956	1	29522.43		
242.2837	1	19240.53		
249.0907	1	122767.83		
250.0938	1	22958.38		
267.1009	1	52670.34		
289.0833	1	43571.73	C17 H14 O3	(M+Na)+
294.1174	1	41010.38		
319.0938	1	20896.53		
437.1934	1	43131.52		

Formula Calculator Element Limits

Element	Min	Max
C	3	60
H	0	120
O	0	30
N	0	10

Formula Calculator Results

Formula	CalculatedMass	CalculatedMz	Mz	Diff. (mDa)	Diff. (ppm)	DBE
C17 H14 O3	266.0943	289.0835	289.0833	0.20	0.69	11.0000

Figure S7: HRMS–ESI spectrum of compound **2**.

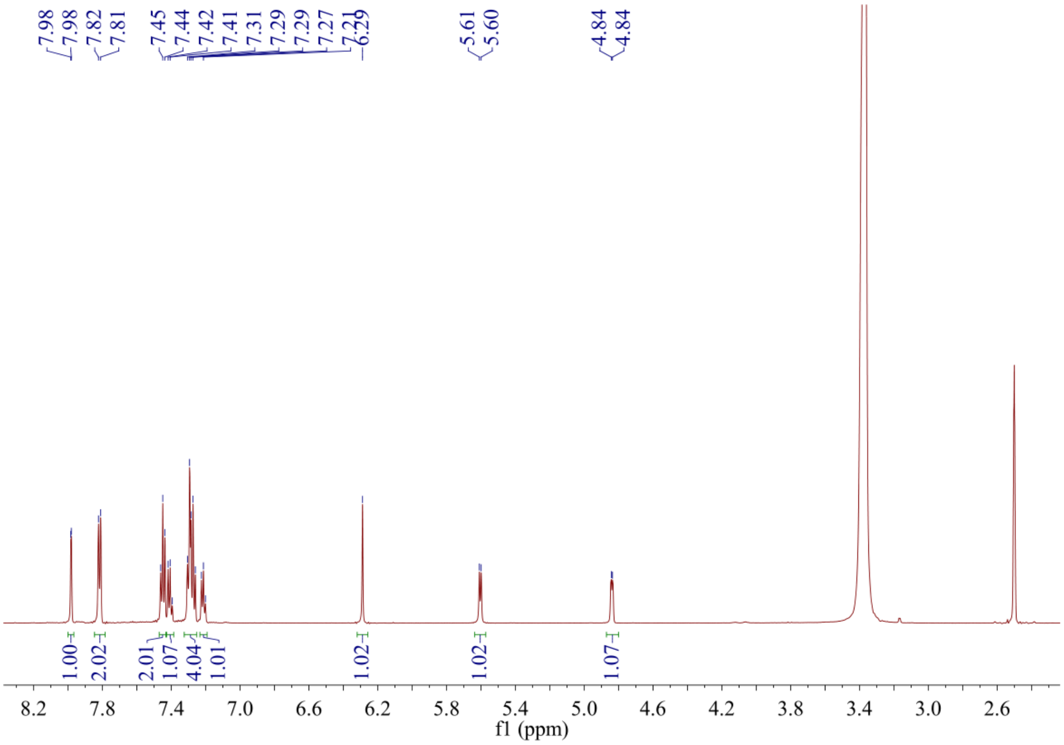

Figure S8: ^{1}H NMR spectrum of compound **2** (600 MHz, DMSO-d_6).

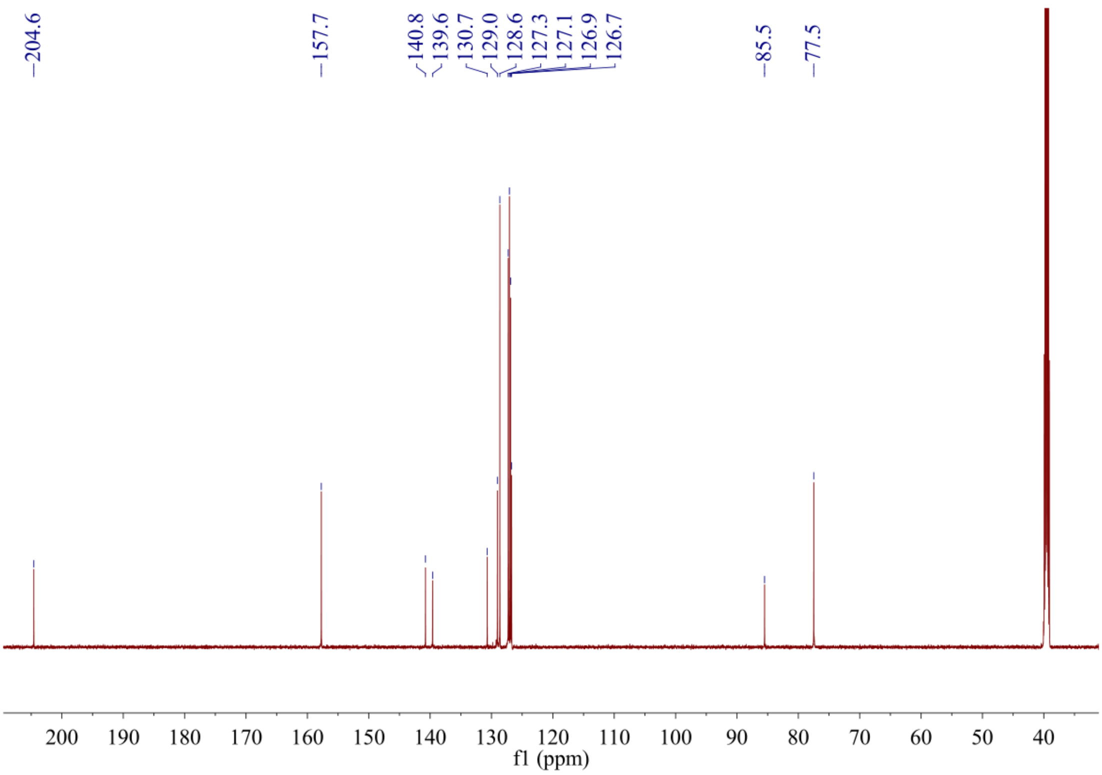

Figure S9: ^{13}C NMR spectrum of compound **2** (150 MHz, DMSO-d_6).

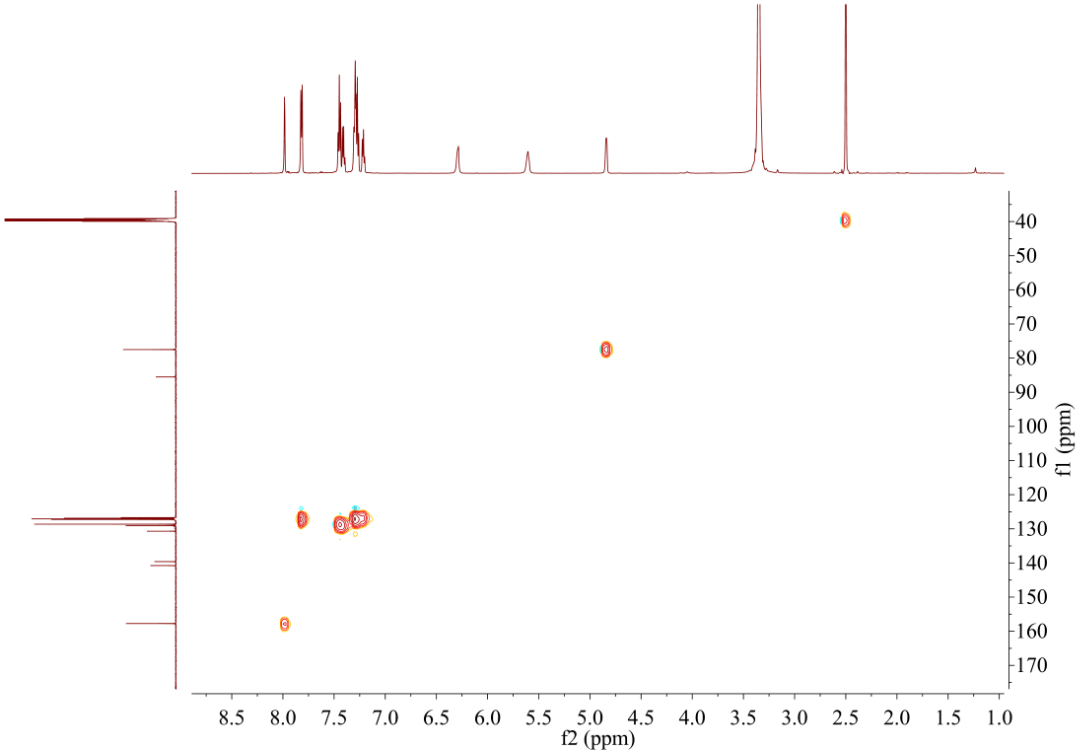

Figure S10: HSQC spectrum of compound **2** (600 MHz, DMSO-d_6).

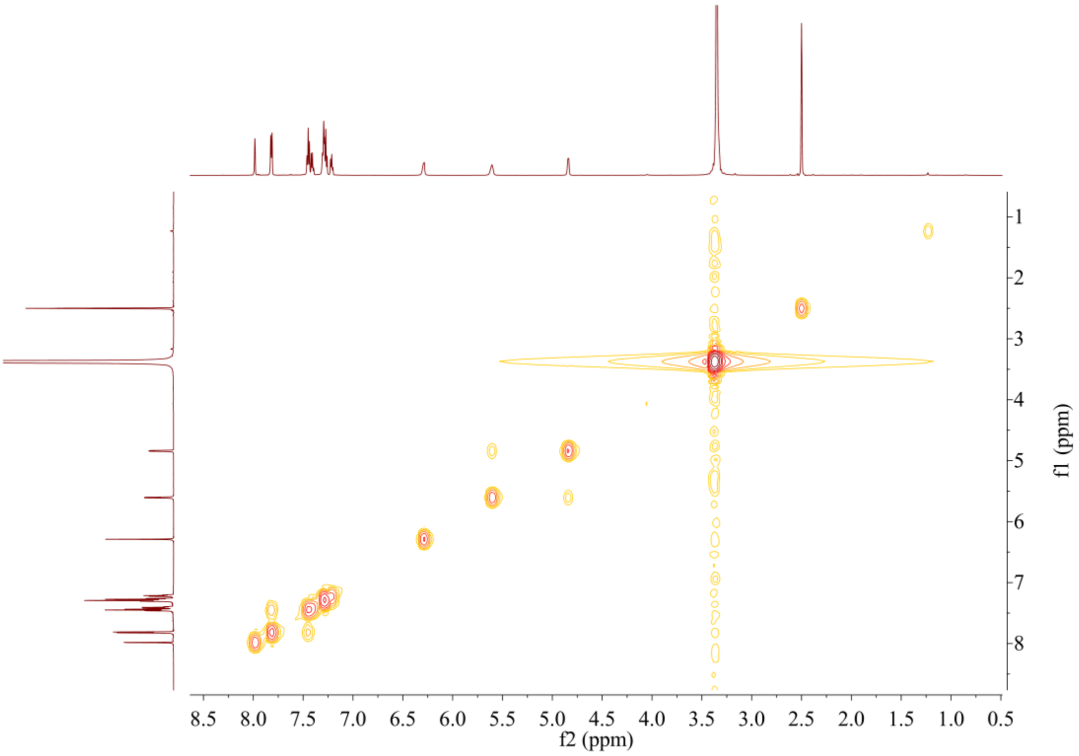

Figure S11: ^{1}H-^{1}H COSY spectrum of compound **2** (600 MHz, DMSO-d_6).

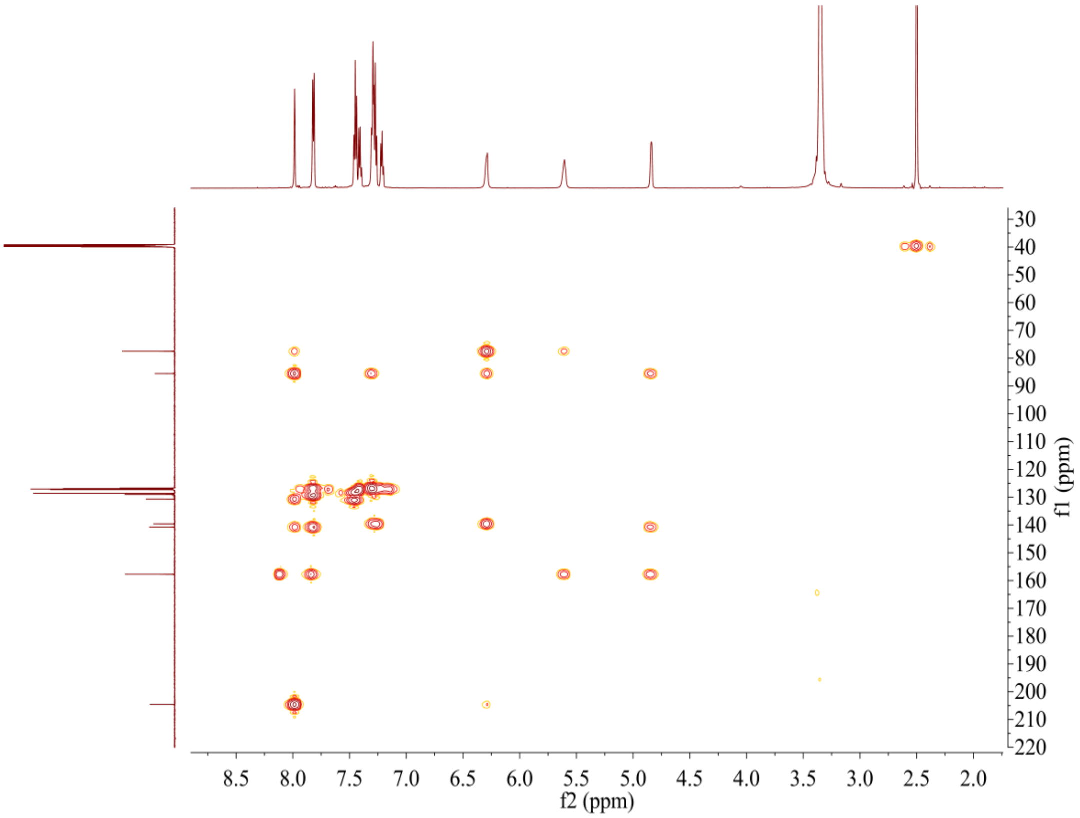

Figure S12: HMBC spectrum of compound **2** (600 MHz, DMSO-d_6).

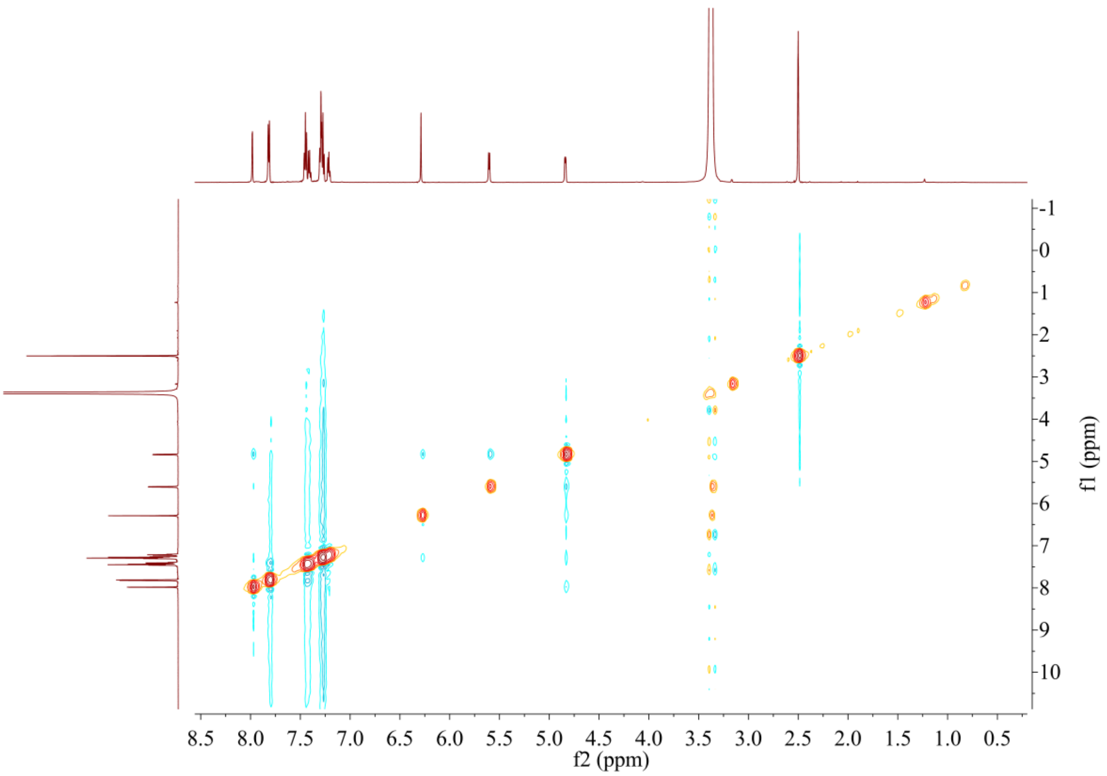

Figure S13: NOESY spectrum of compound **2** (600 MHz, DMSO-d_6).

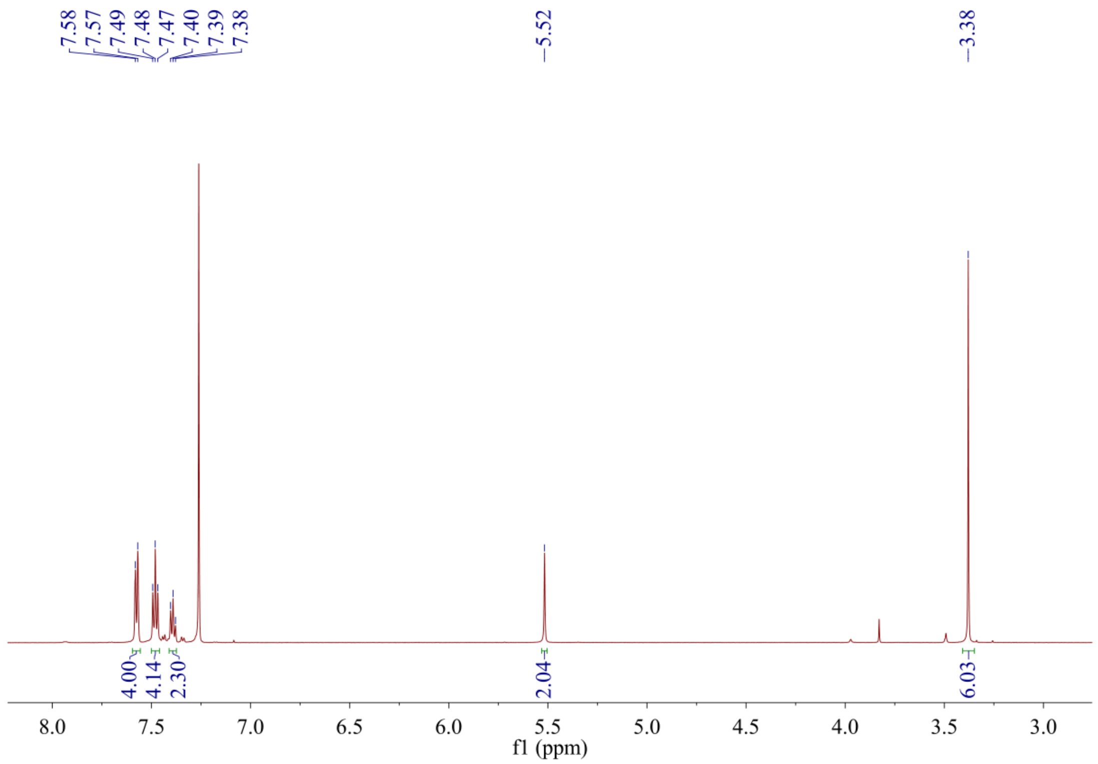

Figure S14: ^{1}H NMR spectrum of compound **3** (600 MHz, $CDCl_3$).

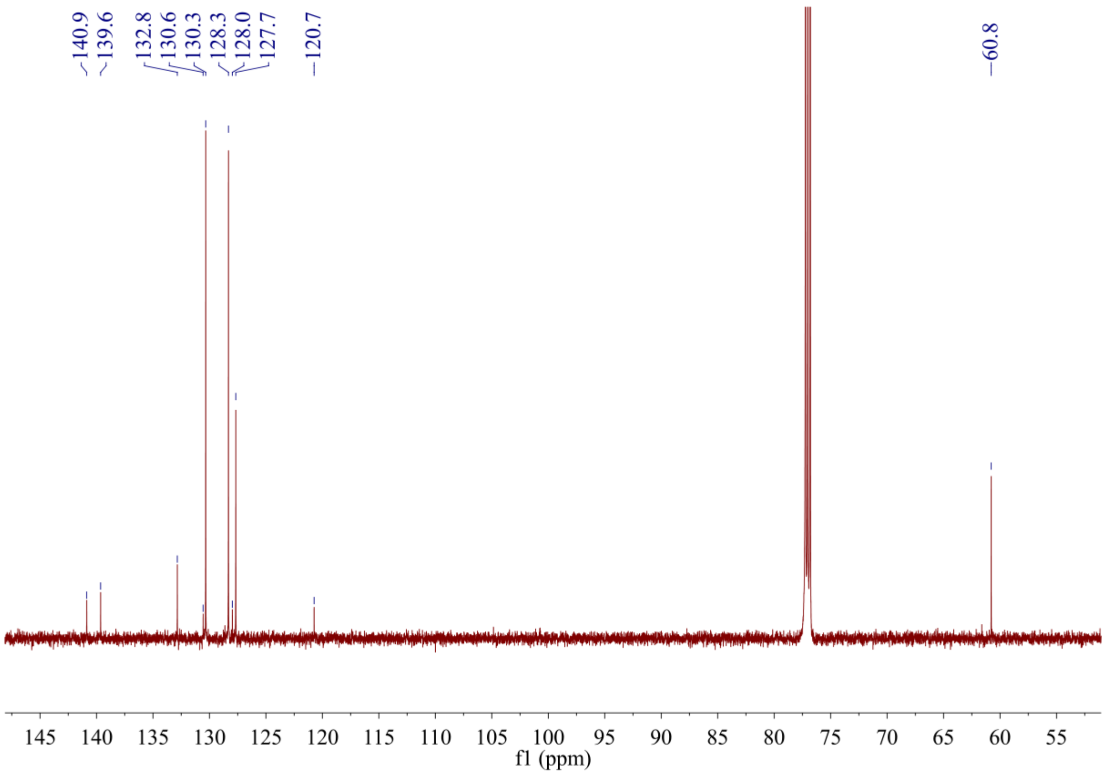

Figure S15: ^{13}C NMR spectrum of compound **3** (150 MHz, $CDCl_3$).

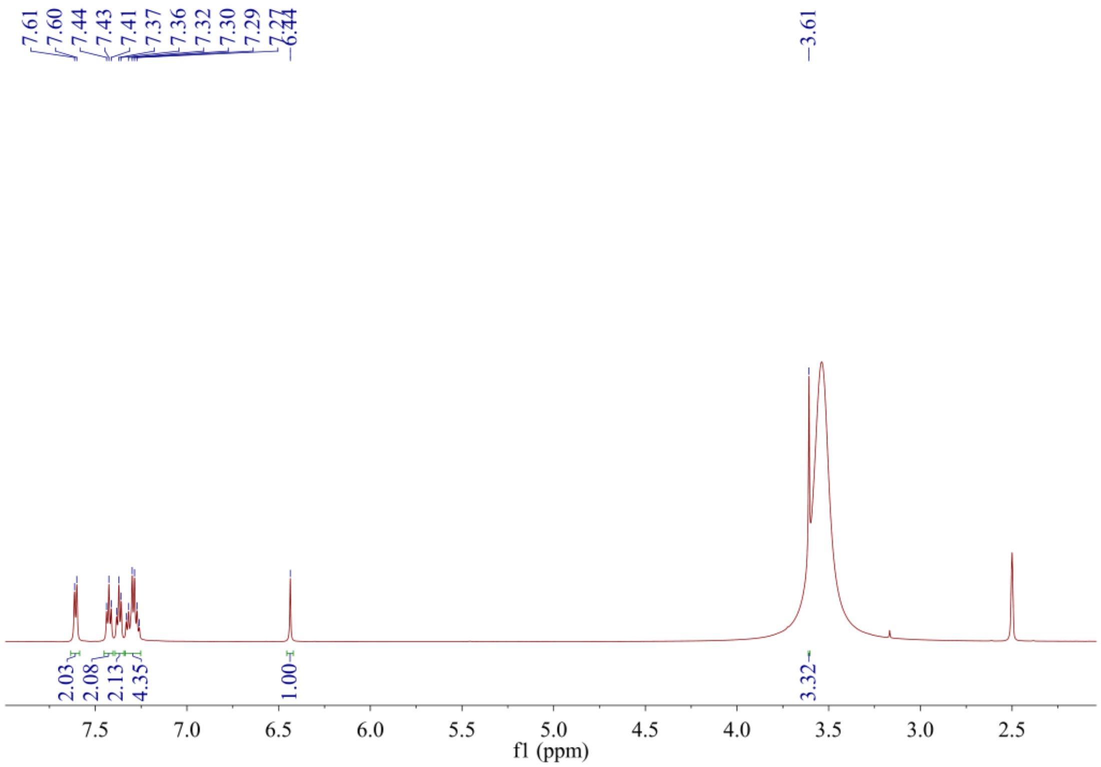

Figure S16: ^{1}H NMR spectrum of compound **4** (600 MHz, DMSO-d_6).

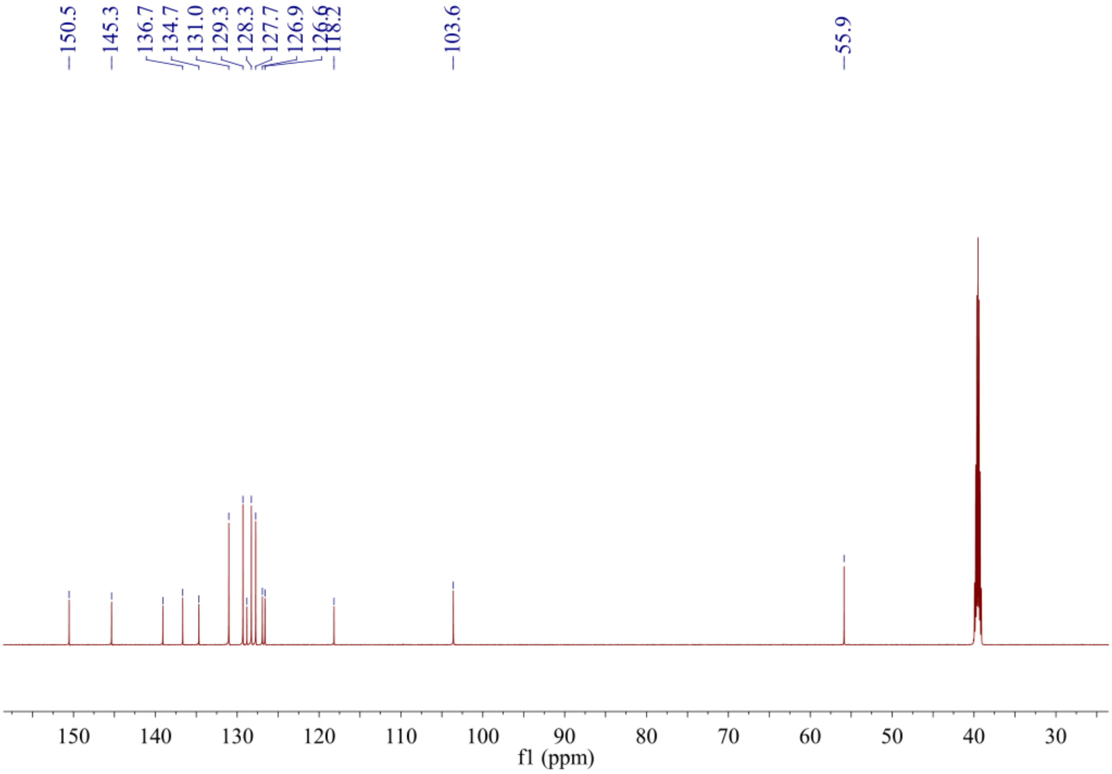

Figure S17: ^{13}C NMR spectrum of compound **4** (150 MHz, DMSO-d_6).

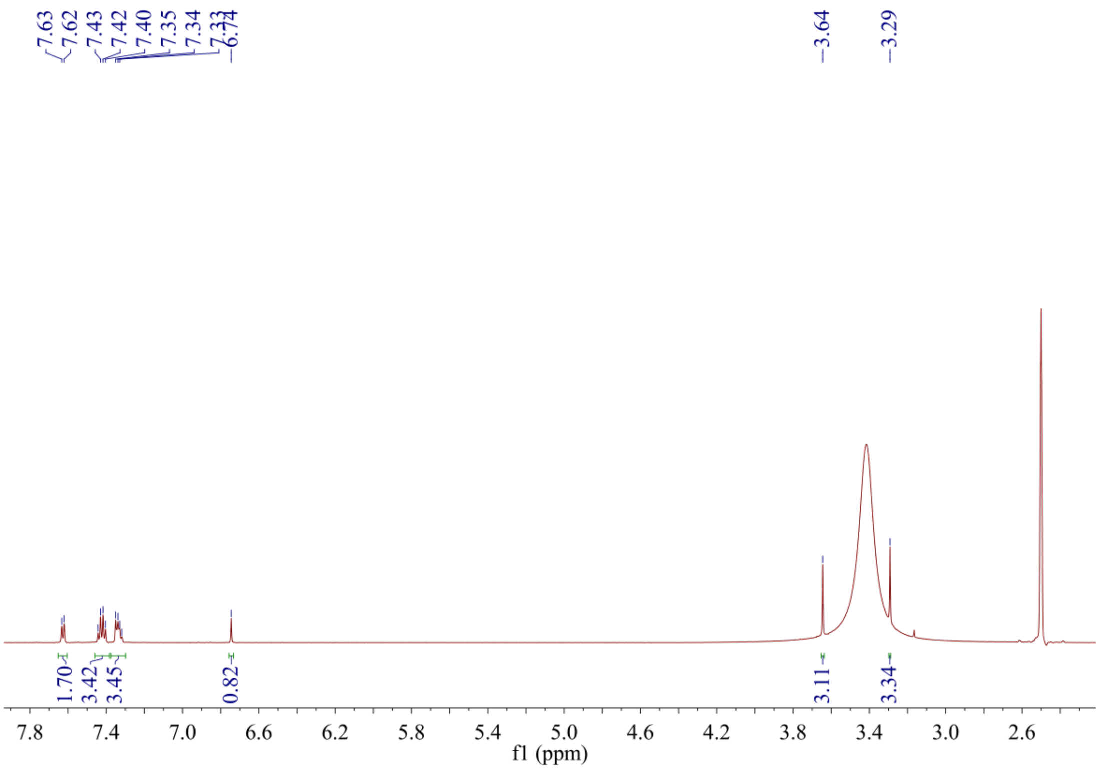

Figure S18: ^{1}H NMR spectrum of compound **5** (600 MHz, DMSO-d_6).

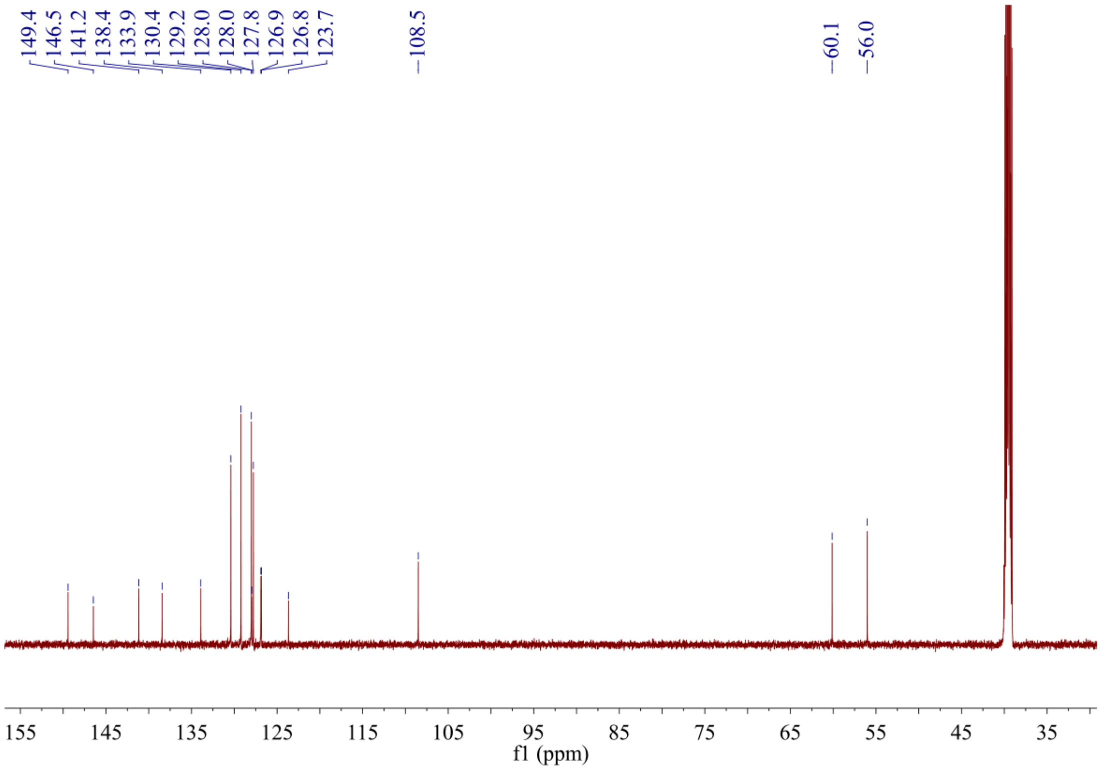

Figure S19: ^{13}C NMR spectrum of compound **5** (150 MHz, DMSO-d_6).

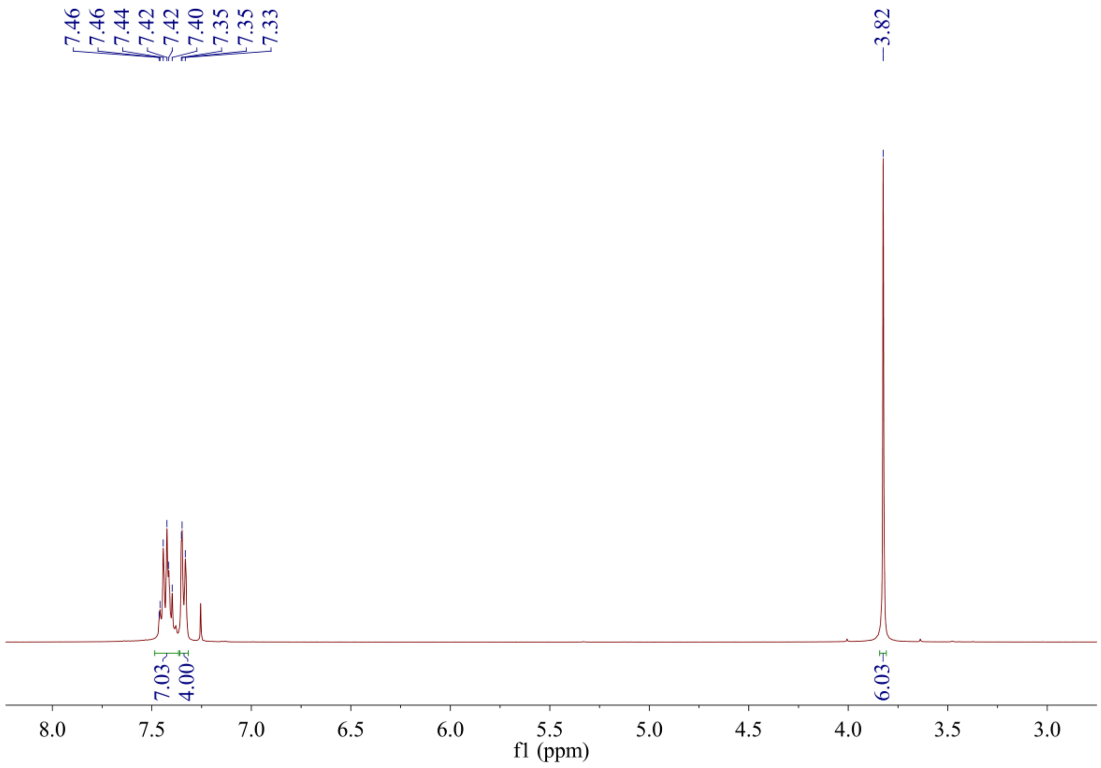

Figure S20: ^{1}H NMR spectrum of compound **6** (600 MHz, $CDCl_3$).

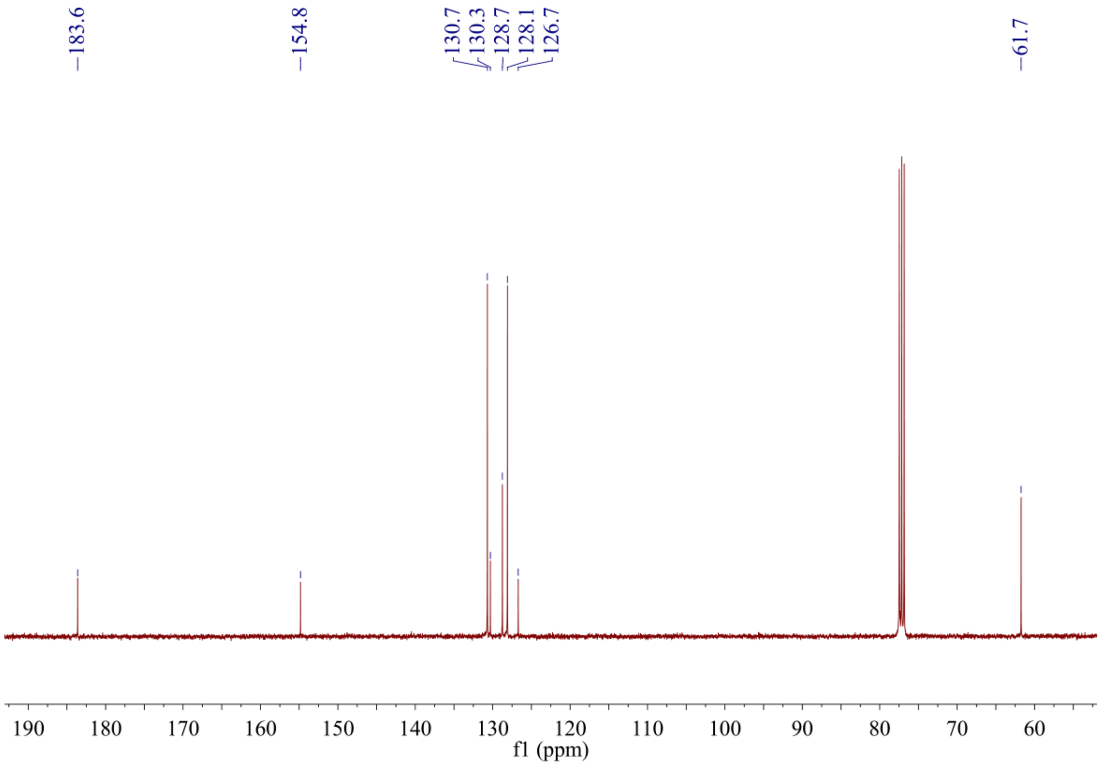

Figure S21: ^{13}C NMR spectrum of compound **6** (150 MHz, $CDCl_3$).

Fragmentor Voltage	Collision Energy	Ionization Mode
135	0	ESI

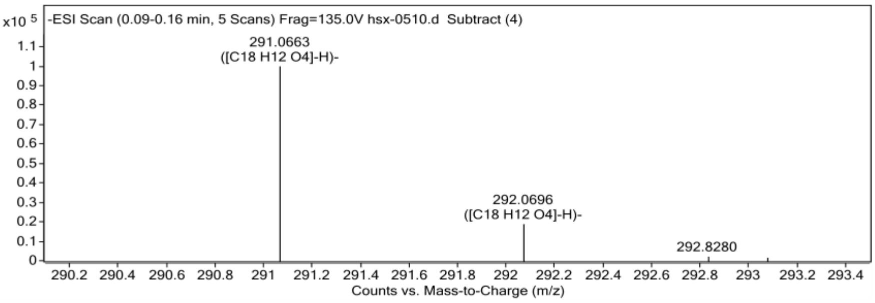

Peak List

m/z	z	Abund	Formula	Ion
91.0555	1	81520.14		
92.928	1	111959.84		
94.9251	1	75297.28		
163.0398	1	93943.83		
268.8008	1	22055.07		
291.0663	1	100265.45	C18 H12 O4	(M-H)-
955.9718	1	569816.38		
956.9743	1	106831.3		
957.9695	1	185085.13		
958.9699	1	32834.93		

Formula Calculator Element Limits

Element	Min	Max
C	3	60
H	0	120
O	0	30

Formula Calculator Results

Formula	CalculatedMass	CalculatedMz	Mz	Diff. (mDa)	Diff. (ppm)	DBE
C18 H12 O4	292.0736	291.0663	291.0663	0.00	0.00	13.0000

Figure S22: HRMS–ESI spectrum of compound **8**.

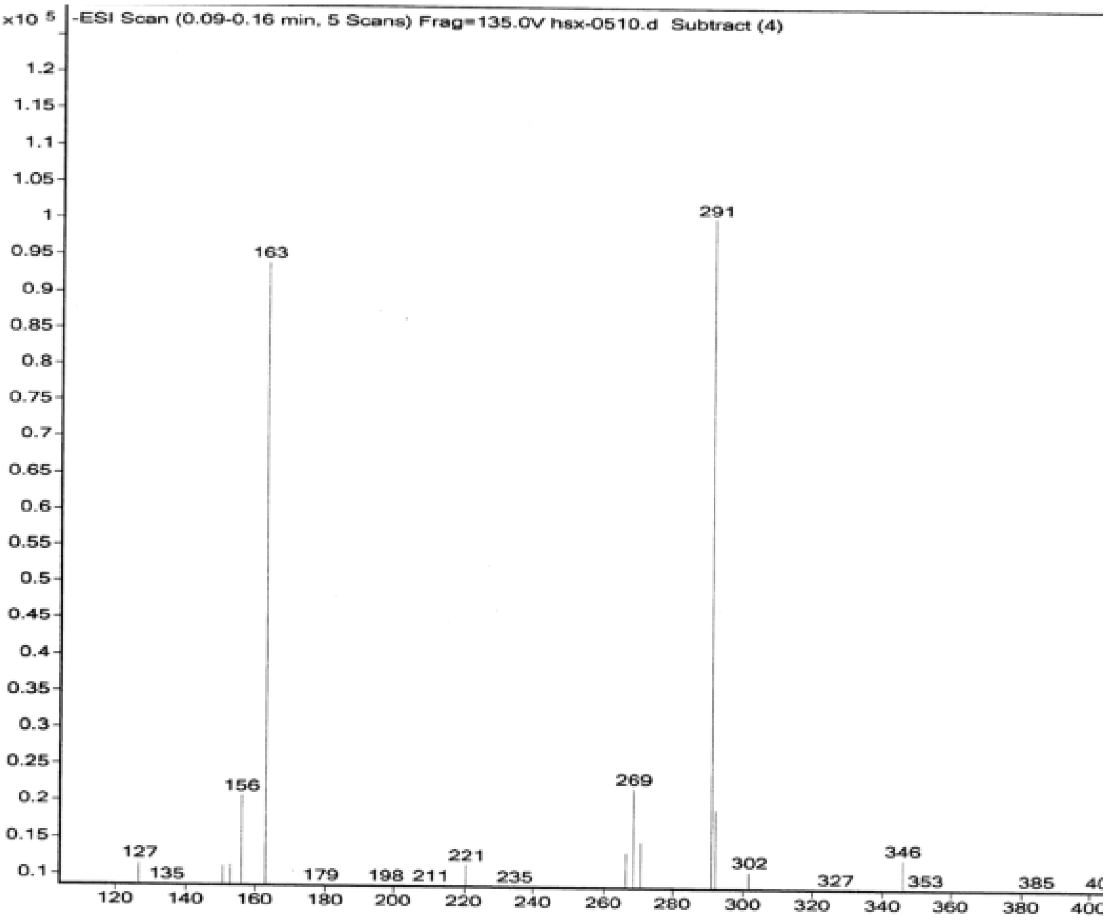

Figure S23: MS analysis of DatA reaction mixture.

Table S1: Annotations of ORFs in the *dat* biosynthetic gene cluster.

ORF	bp/AA	Proposed function	Identity (%)	Accession
datA	2763/920	peptide synthetase	95	KKZ69024.1
datB	927/308	dehydratase	96	KKZ69023.1
datC	405/134	limonene-1,2-epoxide hydrolase	98	KKZ69022.1
Orf1	897/298	hypothetical protein VO63_36430	91	KKZ69021.1
Orf2	681/226	ACP phosphodiesterase	95	KKZ72150.1
Orf3	1368/455	phospho-2-dehydro-3-deoxyheptonate aldolase	96	KKZ72151.1
Orf4	1194/397	chorismate synthase	91	WP_028797313.1
Orf5	315/104	chorismate mutase	84	KKZ72152.1
datD	1443/480	bilirubin oxidase	81	KKZ72238.1
Orf6	366/121	HxlR family transcriptional regulator	99	KKZ72153.1
Orf7	468/155	hypothetical protein VO63_19350	93	KKZ72154.1
datE	930/309	NADPH-dependent oxidoreductase	95	KKZ72155.1

Table S2: Strains and plasmids used and generated in this study.

Stains/Plasmids	Characteristics	Source
Streptomyces		
S. sp. KIB-H1544	Native producer	Isolated
KIB-H1544-Δ*datA*	*datA* inactivation mutant of *S.* sp. KIB-H1544	This study
Escherichia coli		
DH5*α*	Host strain for cloning	Invitrogen
BL21(DE3)	Host strain for expression of protein	Laboratory stock
BW25113/pIJ790	Host strain for PCR targeting	Laboratory stock
ET12567/pUZ8002	Donor strain for conjugation	Laboratory stock
XL1-blue MR	Host strain for genomic library	Agilent Technologies
Plasmids		
pIJ773	Apr^r, Plasmid containing the apramycin resistance gene	Laboratory stock
pSuperCos I-21A4	Kan^r, Cosmid containing *dat* gene cluster used for knockout	This study
p21A4-*ΔdatA*	Apr^r, gene inactivation clone used for mutant *ΔdatA*	This study
pETDuet-sfp	Amp^r, plasmid for the expression of protein	Laboratory stock
pETDuet-sfp-DatA	Amp^r, plasmid for the expression of *datA*	This study
pETDuet-sfp-EchA	Amp^r, plasmid for the expression of *echA*	This study

Abbreviations: Kan^r, kanamycin resistance; Apr^r, apramycin resistance; Amp^r, ampicillin resistance.

Table S3: Primers used in this study.

Target gene	Primer names	Sequences
Primers for screening libraries		
1544SL-1	1544SL-1F	TTCCTGCGACTGGTCTCCCG
	1544SL-1R	CGCCGTTGACGATGATGCTG
1544SL-2	1544SL-2F	CGAAGAACAACGAGGTGTG
	1544SL-2R	CGCTTGAAGTAGAGCATCG
Primer pairs used for mutant producer strain construction		
ΔdatA	datA-F	ATGACCGTGGCCATCCCATCGACCCGGACCGTCGCGGACATTCCGGGGATCCGTCGACC
	datA-R	CCGACATGCCGCGGACGTGGCCGGACGGGGAGTACGTGCTGTAGGCTGGAGCTGCTTC
Primer pairs used for PCR verification of the double cross-over mutant		
ΔdatA	datA-F-YZ	TACTACGCCGAGAGCGACG
	datA-R-YZ	CACCTCGTTGTTCTTCGCG
Primer pairs used for protein expression		
DatA	Duet-DatA-F	cgcggatccgATGACCGTGGCCATCCCAT
	Duet-DatA-R	cgagctcTACTTGCCGTCGCCGAGCG
EchA	Duet-EchA-F	cgcggatccgATGACGTCACAACCCTGGA
	Duet-EchA-R	cgagctcCTATGCCCGGCCCGTGGCC
